# Netrin Signaling Defines the Regional Border in the *Drosophila* Visual Center

**DOI:** 10.1016/j.isci.2018.09.021

**Published:** 2018-09-28

**Authors:** Takumi Suzuki, Chuyan Liu, Satoru Kato, Kohei Nishimura, Hiroki Takechi, Tetsuo Yasugi, Rie Takayama, Satoko Hakeda-Suzuki, Takashi Suzuki, Makoto Sato

**Affiliations:** 1 Mathematical Neuroscience Unit, Institute for Frontier Science Initiative, 13-1 Takaramachi Kanazawa-shi, Ishikawa 920-8640, Japan; 2 Graduate School of Medical Sciences, 13-1 Takaramachi Kanazawa-shi, Ishikawa 920-8640, Japan; 3 School of Medical Sciences, Kanazawa University, 13-1 Takaramachi Kanazawa-shi, Ishikawa 920-8640, Japan; 4 School of Life Science and Technology, Tokyo Institute of Technology, Nagatsuta 4259, Yokohama, Kanagawa 226-8501, Japan

**Keywords:** Neuroscience, Developmental Neuroscience, Mathematical Biosciences

## Abstract

The brain consists of distinct domains defined by sharp borders. So far, the mechanisms of compartmentalization of developing tissues include cell adhesion, cell repulsion, and cortical tension. These mechanisms are tightly related to molecular machineries at the cell membrane. However, we and others demonstrated that Slit, a chemorepellent, is required to establish the borders in the fly brain. Here, we demonstrate that Netrin, a classic guidance molecule, is also involved in the compartmental subdivision in the fly brain. In Netrin mutants, many cells are intermingled with cells from the adjacent ganglia penetrating the ganglion borders, resulting in disorganized compartmental subdivisions. How do these guidance molecules regulate the compartmentalization? Our mathematical model demonstrates that a simple combination of known guidance properties of Slit and Netrin is sufficient to explain their roles in boundary formation. Our results suggest that Netrin indeed regulates boundary formation in combination with Slit *in vivo*.

## Highlights

• Netrin regulates boundary formation in combination with Slit in the fly brain
• Dual Netrin functions as attractant and repellent explain boundary formation

## Graphical Abstract

**Figure:**
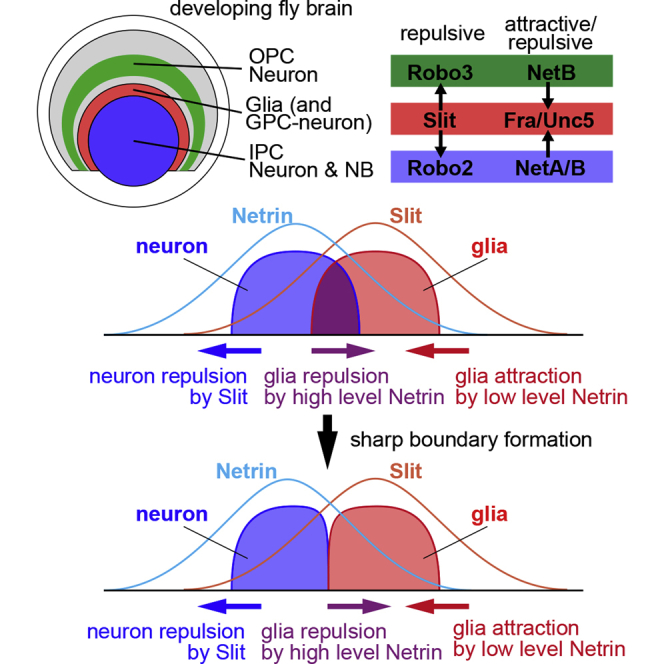

## Introduction

Compartmental subdivision of the brain into each unique region is essential for the development and function of the brain. The established compartmental borders play crucial roles in controlling the behavior of signaling molecules that regulate cell fate as well as in isolating cells in each individual region (Kiecker and Lumsden, 2005, Batlle and Wilkinson, 2012). Although these borders inhibit cell migration across them during development, the inhibition of cell mixing is also crucial for the stabilization of tissue homeostasis in developed organisms because its failure contributes to the accelerated invasion of tumor cells (Abercrombie, 1979, Cortina et al., 2007, Cayuso et al., 2015).

Border formation along each compartment is known to be regulated by three mechanisms: differential affinity in cellular adhesion, interfacial tension between different cell populations, and cell repulsion by intercellular signaling (Batlle and Wilkinson, 2012). Importantly, all of these mechanisms involve molecular machineries located at the cell membrane. In contrast, the mechanism of border formation by diffusible guidance molecules is only poorly understood.

The fly visual center is composed of four ganglia: the lamina, medulla, lobula, and lobula plate. Neurons in these ganglia are mainly derived from two distinct progenitor pools, the outer proliferation center (OPC) and the inner proliferation center (IPC). The neurons in each ganglion are located in specific regions to form sharp compartment boundaries and never intermingle with each other at the interfaces between ganglia. However, the mechanisms that inhibit cell mixing at the borders between ganglia have remained unclear. Previously, we and another group showed that cell-cell interaction through Slit-Robo signaling, a repulsive axon guidance signaling pathway, is involved in the inhibition of cell mixing between the lamina and the IPC (Tayler et al., 2004) and also between the OPC and IPC during larval development (Suzuki et al., 2016). However, because the cell mixing occurs only partially even by severe disruption of Slit-Robo signaling, additional signaling pathways likely also participate in the formation of these borders.

Here, we show that Netrin signaling, another axon guidance signaling pathway, regulates the formation of the border between the OPC and IPC. The ligands Netrin A (NetA) and Netrin B (NetB) are expressed in the IPC, whereas their receptors Frazzled (Fra, *Drosophila* homologue of Deleted in colorectal cancer [DCC]) (Kolodziej et al., 1996) and Unc5 are expressed in lamina glial cells located at the border between the OPC and IPC. In the *NetA* and *NetB* double mutant (*NetAB*), *fra* or *unc5* mutant, IPC cells intruded into the OPC across the compartmental border. Since Fra and Unc5 are expressed in the lamina glial cells, we examined glia-specific loss of function of Fra and Unc5, which also caused cell mixing, suggesting that glial cells play essential roles in the formation of the border between the OPC and IPC through Netrin signaling.

It has been suggested that Fra acts as an attractive receptor, whereas Unc5 acts as a repulsive receptor of Netrin ligands (Kolodziej et al., 1996, Keleman and Dickson, 2001, Brankatschk and Dickson, 2006, Timofeev et al., 2012). In addition, it has also been suggested that a low level of Netrin acts as an attractant, whereas a high level of Netrin acts as a repellent when it is received by neurons expressing both DCC and Unc5 (Taylor et al., 2015). Because it is hard to imagine that these guidance molecules are regulating border formation, we formulated a mathematical model by simply combining the dual function of Netrin and repulsive action of Slit. Interestingly, our model demonstrated that the guidance functions of Netrin and Slit are sufficient to explain their roles in boundary formation. Since these signaling pathways are evolutionarily conserved from insects to mammals, their roles in establishing the tissue border may also be conserved across species.

## Results

### Netrin and Its Receptors Are Expressed in Each Domain of the Optic Lobe during Larval Development

**Figure 1:**
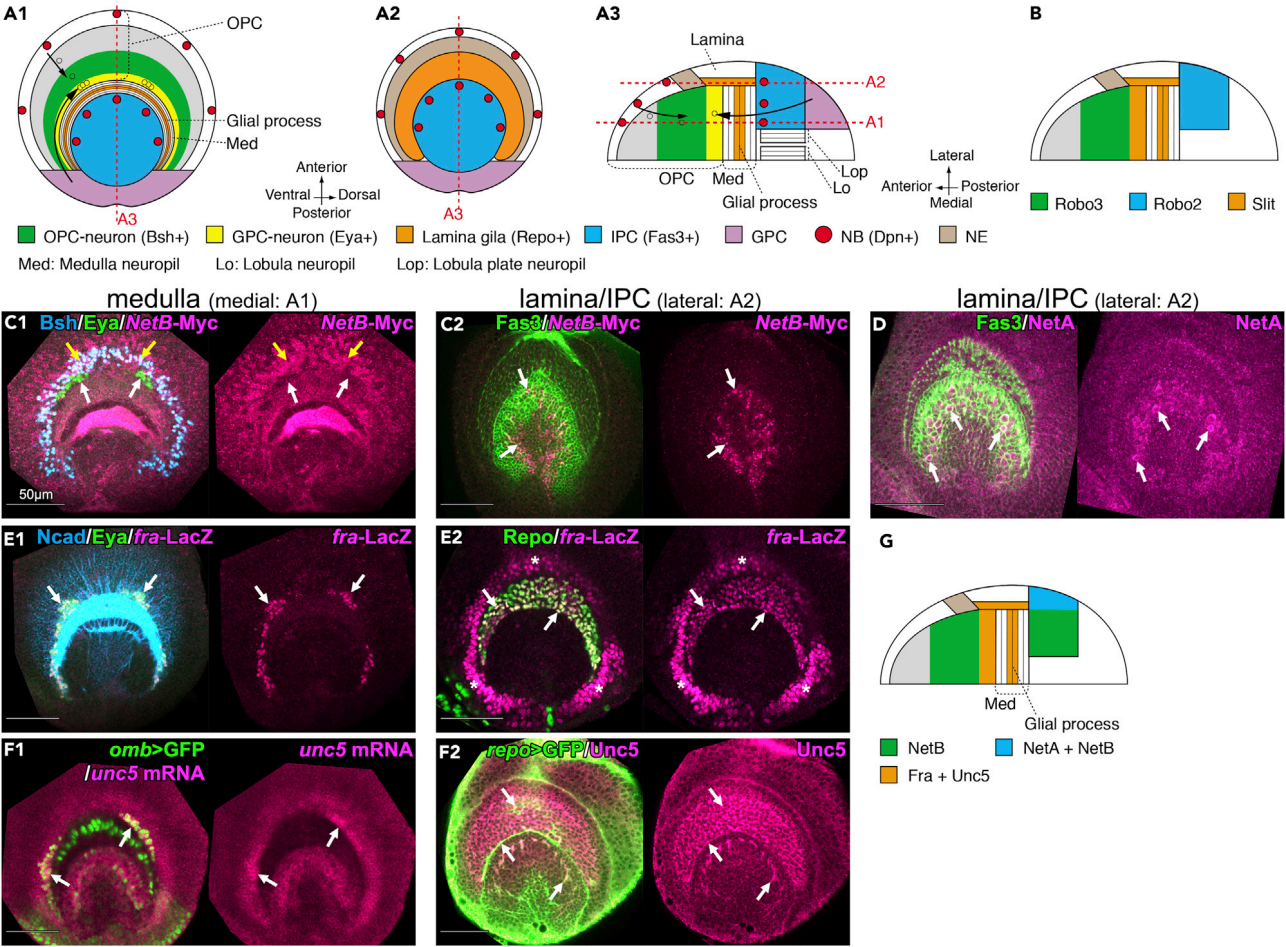
Expression Patterns of Netrin Ligands and Their Receptors in the Visual Center (A) Schematics of the larval medulla primordium in lateral (A1-2) and horizontal views (A3). Medial (A1) and lateral sections (A2) are shown (red dotted lines in A3). OPC-NBs (red) produce medulla neurons in a linear and radial orientation toward the center of medulla (green, small arrows). GPC-neurons migrate tangentially and are placed in the innermost region of OPC (yellow, large arrows). Lamina glia (orange) is found in the lateral section (A2) and project glial processes inside the medulla neuropil (A3). (B) Expression patterns of Slit (orange), Robo2 (blue), and Robo3 (green). (C–F) Lateral views of the developing optic lobe at late third instar larval stage. (C1, E1, and F1). Medial sections showing the medulla as indicated in (A1). (C2, D, E2, and F2) Lateral sections showing the lamina and IPC as indicated in (A2). Localizations of NetA, NetB, Fra, and Unc5 are shown. (C1) *NetB-*Myc (magenta) is localized in the Bsh+ neurons (blue) as indicated by yellow arrows but not in the Eya+ neurons (green) as indicated by white arrows. (C2) Both *NetB-*Myc (magenta) and (D) NetA (magenta) are also localized in a subset of IPC cells (Fas3+; green) as indicated by arrows. (E) *fra-*LacZ (magenta) is expressed in Eya+ neurons (green in E1), lamina glial cells (Repo+; green in E2) as indicated by arrows, and putative neuroepithelial cells (asterisks; NE in A2). (F) *unc5* mRNA (magenta in F1) is expressed in Eya+ neurons (visualized with *omb-Gal4 UAS-nlsGFP*; green in F1) as indicated by arrows, and Unc5 is localized in lamina glial cells (visualized with *repo-Gal4 UAS-CD8GFP* in F2). (G) A schematic of the expression patterns of NetA, NetB, Fra, and Unc5. Both NetA and NetB are localized in the lateral IPC (blue). NetB is exclusively accumulated in medial IPC and OPC neurons (green). Both Fra and Unc5 (orange) are localized in the lamina glial cells and GPC neurons.

We have previously reported that Slit-Robo signaling is important for the proper arrangement of medulla neurons by establishing the border between the OPC and IPC (Figure 1A) (Suzuki et al., 2016). This border was not completely disrupted in *slit*, *robo3*, or *robo2* mutants, suggesting that other signaling pathways are also involved in the border formation. To identify other regulatory signaling pathways, we conducted expression screening for typical axon guidance molecules and found that Netrin and its receptors are expressed in the medulla primordium. First, we examined the localization patterns of the ligands NetA and NetB. NetA localization was exclusively found in a subset of the lateral IPC cells (Figures 1D and 1G, white arrows), whereas NetB-myc was localized in the Bsh+ OPC-derived neurons (Figure 1C1, yellow arrows) and in a subset of the lateral IPC cells (Fas3+) located next to the lamina (Figure 1C2, white arrows) (Brankatschk and Dickson, 2006). Next, we examined expression patterns of Netrin receptors using *fra-LacZ*, an enhancer trap line for *fra*, *in situ* hybridization for *unc5* mRNA, and antibodies against Fra and Unc5. *fra-LacZ* was expressed in the glia precursor cell (GPC)-derived neurons (Eya+) that are located in the innermost area of the OPC (Figure 1E1, white arrows) and also in the lamina glial cells (Repo+; Figure 1E2, white arrows). The *fra-LacZ*-positive cells near the lamina glial cells are most likely neuroepithelial cells in the OPC (Figure 1E2, asterisks). We also observed Fra protein localization in the lamina glial cells (data not shown). The localization pattern of Unc5 protein was quite similar to that of Fra, with localization in the lamina glial cells visualized using *repo-Gal4 UAS-CD8GFP* (Figure 1F2; white arrows). Note that *repo-Gal4 UAS-CD8GFP* visualizes the glial cell membrane, whereas Repo antibody visualizes the glial cell nuclei, because Repo is a nuclear protein. As observed in *fra-LacZ*, *unc5* mRNA was also expressed in the GPC-derived neurons visualized by *omb-Gal4 UAS-nlsGFP* (Figure 1F1; white arrows; a subset of GFP-positive cells). Taken together, Netrin ligands are expressed in neurons derived from IPC and OPC, whereas both of their receptors are expressed in the GPC-derived neurons and in lamina glial cells (Figure 1G). The lamina glia cells project their processes inside the medulla neuropil as discussed later (Figures 3F and 3G).

### Netrin Signaling Regulates the Formation of the Border between OPC and IPC

**Figure 2:**
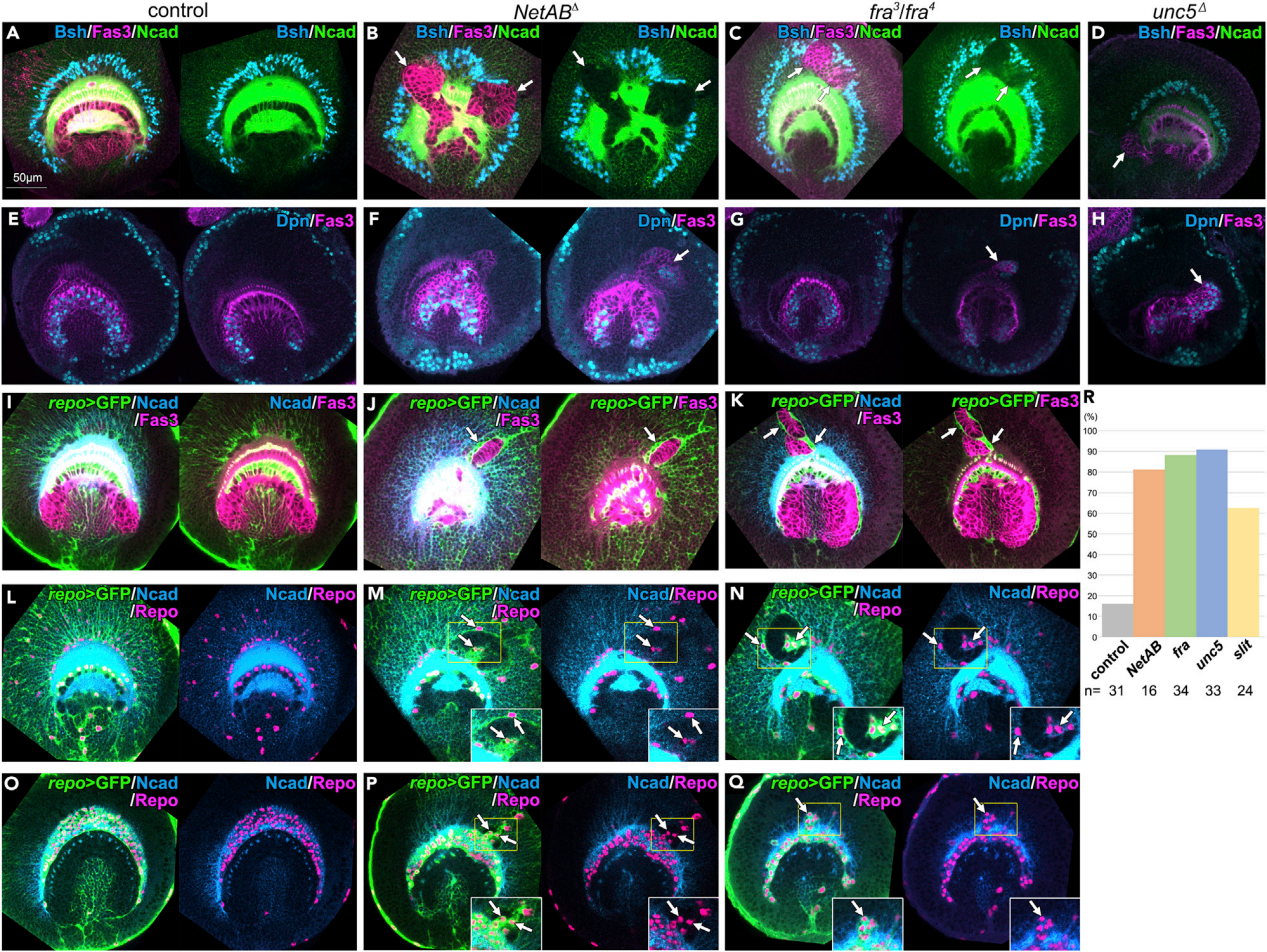
Netrin Signaling Regulates the Border Formation in the Optic Lobe (A–Q) Lateral views of the developing medulla (A–N, medial sections) and lamina (O–Q, lateral sections) at the late third instar larval stage. (A, E, I, L, and O) Control brains. (A–H) In *NetAB*^*Δ*^ (B and F), *fra*^*3*^*/fra*^*4*^ (C and G), and *unc5*^*Δ*^ mutants (D and H), ectopic IPC cells (Fas3+; magenta) are found in OPC (arrows). (A–D) The arrangement of Bsh+ OPC neurons and Ncad+ neuropile are disrupted. (E–H) Ectopic NBs (arrows, Dpn+; blue) are found in ectopic IPC cells (Fas3+; magenta). Lateral and medial regions are shown in left and right panels, respectively (E–G). (I–Q) Distribution of glial cells is visualized with *repo-Gal4 UAS-CD8GFP* (green) in control (I, L, and O), *NetAB*^*Δ*^ (J, M, and P), and *fra*^*3*^*/fra*^*4*^ (K, N, and Q). (I–K) ectopic IPC cells (Fas3+; magenta) are enwrapped with glial processes (arrows). (L–Q) ectopic glial cells (Repo+; magenta) are ectopically observed as indicated by white arrows in OPC (L–N) and lamina (O–Q). (R) Boundary defects in control, *NetAB*^*Δ*^, *fra*^*3*^*/fra*^*4*^*, unc5*^*Δ*^, and *slit*^*dui*^*/slit*^*2*^ mutant brains are quantified and statistically tested by Fisher's exact test (p < 0.0005).

To reveal the roles of Netrin signaling, we first examined the effects of *NetA* and *NetB* mutations on the arrangement of OPC and IPC cells (Suzuki et al., 2016). Throughout this study, Bsh, Fas3, and Ncad antibodies were used to visualize OPC-derived neurons, IPC cells, and the neuropil structure, respectively. IPC cells penetrated the OPC region and suppressed the formation of neuropil structures in *NetAB*^*Δ*^ brains (Figures 2A, 2B, and 2R). At the same time, distribution of OPC-derived neurons was disrupted. However, we have never observed penetration of OPC cells into the IPC region. Ectopic IPC cells were only rarely observed in *NetA*^*Δ*^ and *NetB*^*Δ*^ larvae, suggesting that NetA and NetB act redundantly (data not shown). These results suggest that Netrin plays important roles in the establishment of the boundary between OPC and IPC that restricts IPC cells within IPC during larval development. Interestingly, the cells that usually express Netrins were misplaced in *NetAB* mutant brains, suggesting that the cells that express Netrin receptors play important roles in boundary formation.

Therefore, we examined contributions of Fra and Unc5 receptors by analyzing loss-of-function mutants. In *fra*^*3*^*/fra*^*4*^ larvae, the IPC cells invaded the OPC (Figures 2C and 2R) and the distributions of OPC-derived neurons were dramatically disrupted, as observed in *NetAB*^*Δ*^ larvae. Similar defects were observed in *unc5* null mutant brains (Figures 2D and 2R). Again, invasion of the OPC cells into the IPC region was never observed. Thus, these results suggest that Netrin signaling plays important roles in establishing the boundary between the OPC and IPC to prevent IPC cells from penetrating the OPC. Note that Fas3+ ectopic IPC cells contain neuroblasts (NBs), which extensively proliferate (Figures 2F–2H). These ectopic NBs may derive from IPC, because there are many IPC NBs near the boundary between the IPC and OPC in control brains (Figure 2E). Consistent with the observation that the OPC cells do not penetrate the IPC, there is no OPC NB near the OPC-IPC boundary. The Fas3+ IPC NBs may penetrate the OPC destroying the boundaries in the mutant brains. We did not find any positional bias in the above-mentioned defects. The invasion of IPC cells equally occurred in the ventral, dorsal, and central part of the brain.

The area of the ectopic IPC cells found in the OPC region was extremely variable, which might be related to the timing of IPC NB penetration during development. Therefore, we simply compared the numbers of brain samples showing the boundary defect (Figure 2R).

We also found that ectopic IPC cells were frequently surrounded by glial cells visualized with *repo-Gal4 UAS-CD8GFP* in *NetAB*^*Δ*^ and *fra*^*3*^*/fra*^*4*^ mutants (Figures 2I–2K; n = 29/29 and 22/25, respectively), suggesting that the arrangement of glial cells is disrupted in these mutants. We also examined the distribution of glial nuclei in the mutant medulla and found that glial cells (Repo+) appeared within and around Ncad-negative regions in *NetAB*^*Δ*^ and *fra*^*3*^*/fra*^*4*^ mutants (Figures 2L–2N; n = 26/49 and 9/25, respectively). We examined the distribution of glial cells in the lamina as well as in the medulla primordium in these mutants because lamina glial cells are thought to be important for the formation of the border by acting as a source of Slit (Tayler et al., 2004). Glial cells were always observed within the Ncad+ lamina plexus region in the controls (Figure 2O). However, a subset of lamina glial cells was ectopically found outside the lamina plexus in *NetAB*^*Δ*^ and *fra*^*3*^*/fra*^*4*^ mutants (Figures 2P and 2Q; n = 18/45 and 16/28, respectively). Thus, these results suggest that Netrin signaling controls the distribution of glial cells.

### Glia-Specific Inhibition of Netrin Signaling Disrupts the Border between the OPC and IPC

**Figure 3:**
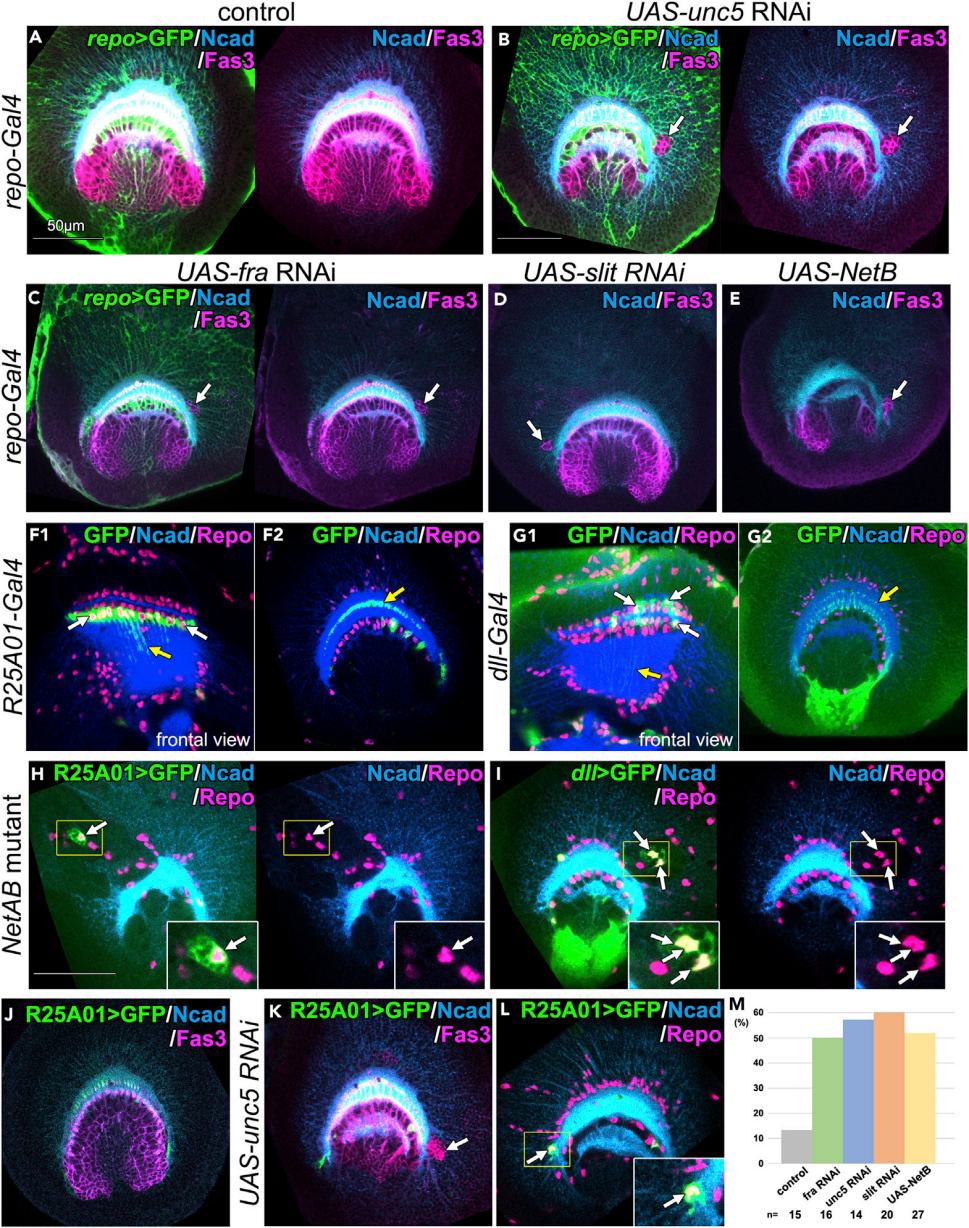
Suppression of Netrin Signaling in Glial Cells Disrupts the Border Formation (A–E, F2, G2, and H–L) Lateral views of the developing medulla at the late third larval instar stage (medial sections). (A–E) Ectopic IPC cells (Fas3+; magenta, arrows), neuropil structure (Ncad+; blue), and glial cell membrane (UAS-CD8GFP+); green in (A–C) are compared. (A) Control. (B–D) *unc5, fra*, and *slit* knock down under the control of *repo-Gal4*, respectively. (E) Ectopic *NetB* expression under the control of *repo-Gal4*. (F–L) Lamina glial cells are visualized by (F, H, and J–L) *R25A01-Gal4 UAS-CD8GFP* and (G and I) *dll-Gal4 UAS-CD8GFP* (arrows). Frontal (F1 and G1; anterior view of Figure 1A3 focusing on glial process) and lateral views (F2, G2; see Figure 1A1), in which the processes of lamina glial cells are found within the medulla neuropil (yellow arrows) (F1 and G1) lateral to the top. (H and I) In *NetAB* mutant, ectopic lamina glial cells co-expressing Repo (magenta) are observed (arrows). (J–L) *unc5* RNAi under the control of *R25A01-Gal4 UAS-CD8GFP* (green) induces ectopic IPC cells (K; Fas3+; magenta; arrow) and ectopic glial cells (L; Repo+; magenta; arrows). (J) Control. (M) Frequency of samples showing the boundary defect is compared in control, *fra* RNAi, *unc5* RNAi, *slit* RNAi, and *NetB* ectopic expression under the control of *repo-Gal4*. Examined sample numbers are shown at the bottom. Statistically tested by Fisher's exact test (p < 0.03).

As observed in Figure 1, Netrin receptors are expressed in the lamina glial cells in addition to the GPC-derived neurons. Moreover, Netrin signaling dysfunction resulted in ectopic appearance of Fas3+ IPC cells surrounded by glial cells in the OPC (Figures 2I–2K). These observations raise a possibility that Netrin signaling is activated in lamina glial cells to regulate the border formation. To examine this possibility, we conducted glial-cell-specific suppression of Netrin signaling using RNAi lines against *fra* and *unc5*. We induced *fra* RNAi and *unc5* RNAi under the control of *repo-Gal4* and observed ectopic appearance of Fas3+ IPC cells in the medulla primordium (Figures 3A–3C and 3M). A similar result was obtained by *slit* RNAi (Figures 3D and 3M; *slit*^*JF01228*^, n = 11/31; *slit*^*JF01229*^ n = 12/20; *slit*^*GD5822*^, n = 7/10), indicating that Netrin signaling as well as *slit* expression in glial cells is essential for the formation of the border between the OPC and the IPC. Ectopic *NetB* expression in glial cells also caused a similar boundary defect (Figures 3E and 3M), suggesting that Netrin ligand expression needs to be restricted to the OPC-derived neurons and IPC cells. The numbers of brain samples showing the boundary defect were compared (Figure 3M).

There are three layers of lamina glial cells, namely, epithelial, marginal, and medulla glia (Poeck et al., 2001). To examine which types of lamina glial cells regulate the border formation, we used two Gal4 drivers expressed in different subsets of lamina glial cells, *R25A01-Gal4* (Edwards et al., 2012) and *dll-Gal4*. *R25A01-Gal4* is exclusively expressed in the medulla glia, the third glial sheath located between the lamina and the medulla (Figure 3F, arrows), whereas *dll-Gal4* is expressed in both epithelial and marginal glia, the first and second glial sheaths, respectively (Figure 3G, arrows). These cells project long glial processes inside the medulla neuropil (yellow arrows in Figures 3F and 3G) (Poeck et al., 2001). First, we examined the distribution of lamina glial cells in the *NetAB*^*Δ*^ mutant and found that *R25A01-Gal4*+ and *dll-Gal4*+ glial cells visualized with GFP ectopically appeared in the OPC (Figures 3H and 3I, arrows; n = 13/72 and 12/58, respectively). Next, we knocked down Netrin signaling in lamina glial cells. Induction of *unc5* RNAi under the control of *R25A01-Gal4* resulted in ectopic Fas3+ IPC cells (Figures 3J and 3K, arrows). Ectopic Fas3+ IPC cells were observed in the OPC (*unc5*^*KK102074*^, n = 26/37 and *unc5*^*GD3510*^, n = 15/25), and *R25A01-Gal4*+ glial cells appeared ectopically in the OPC (Figure 3L; *unc5*^*KK102074*^, n = 5/12 and *unc5*^*GD3510*^, n = 9/25). Although *unc5* RNAi under the control of *dll-Gal4* caused boundary defects (data not shown), *dll-Gal4* is weakly expressed throughout the optic lobe (Figure 3G). We need to use Gal4 drivers that are specifically expressed in the epithelial and/or marginal glia to clarify their roles. However, the aforementioned results suggest that Netrin signaling in lamina glial cells regulate the border formation.

### Mathematical Modeling of Boundary Formation by Slit and Netrin

**Figure 4:**
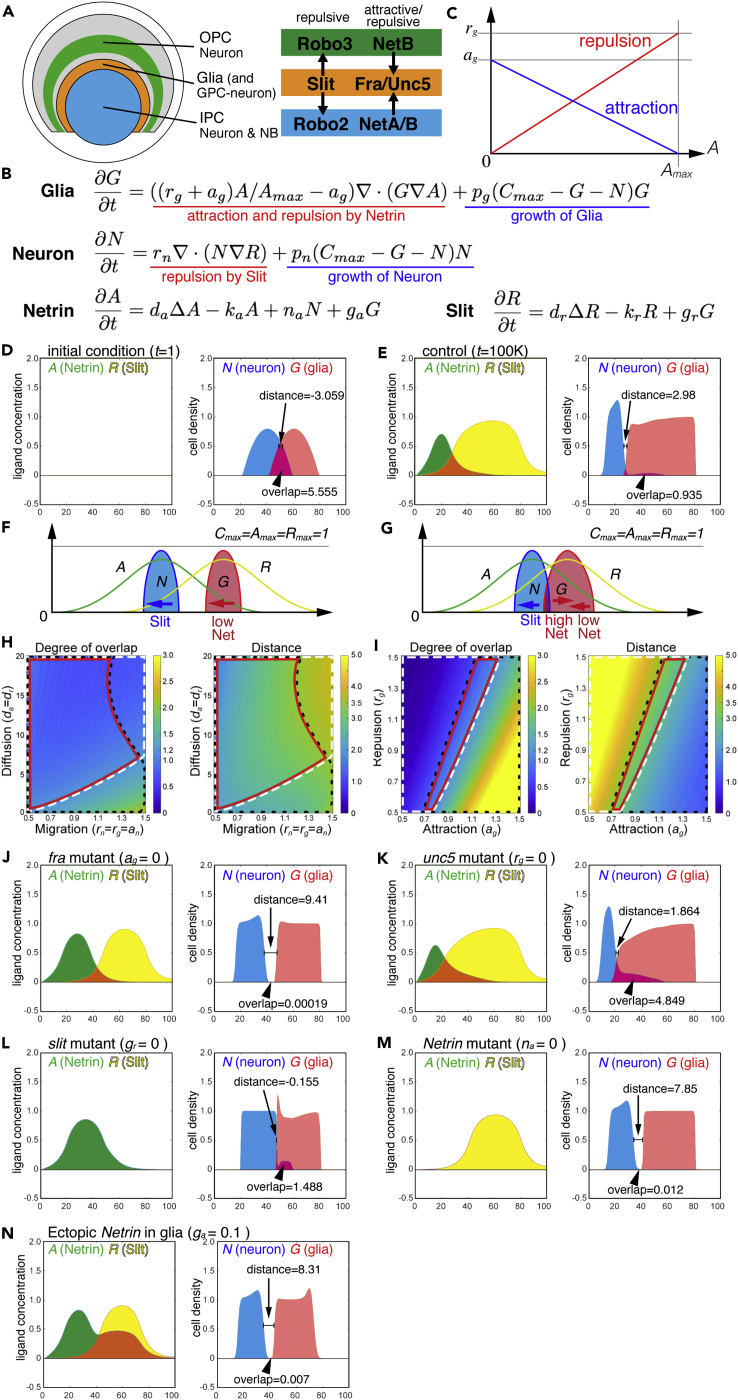
Mathematical Modeling of the Boundary Formation by Slit and Netrin Signalings (A) Simplified drawing of the expression patterns of Slit, Robo, Netrin, Fra, and Unc5 in the larval optic lobe. In our model, Slit, Fra, and Unc5 are expressed in glia, whereas Robo2/3 and NetA/B are expressed in neurons. (B) The mathematical model including four variables *G* (glia), *N* (neuron), *A* (Netrin), and *R* (Slit). (C) The attraction and repulsion of glia by different concentrations of Netrin. (D–N) Numerical results: *A* (Netrin; green), *R* (Slit; yellow), *N* (neuron; blue), and *G* (glia; red). Orange and magenta indicate the overlaps of Netrin and Slit, and neuron and glia, respectively. The *x* axis indicates a one-dimensional space (D–G and J–N). (D) Initial condition at *t* = 1. (E and J–N) Results at t = 100,000. (E) Control (*a*_*g*_ = *r*_*g*_ = *r*_*n*_ = 1). (F and G) Boundary formation by dual action of Netrin. When the glial cell cluster is distant from the neuron cluster, glia is attracted to neuron by the gradient of low Netrin concentration (F). When the glial cell cluster overlaps the neuron cluster, glia is repelled by neuron due to high Netrin concentration at the interface (G). (H and I) Phase diagrams showing the degree of overlap (left panels; 0–3.0) and distance between neuron and glia (right panels; 0–5.0). The areas with less overlap (less than 1.2 based on the left panels) and less distance (less than 3.5 based on the right panels) are indicated by white and black broken lines, respectively. Red lines indicate the overlap between the white and black lines showing the conditions for sharp boundary. (H) Diffusion of *A* and *R* (*d*_*a*_ = *d*_*r*_) is changed between 5 and 20, whereas migration of *N* and *G* (*r*_*n*_ = *r*_*g*_ = *a*_*g*_) are changed between 0.5 and 1.5. (I) Attraction and repulsion of *G* (*a*_*g*_ and *r*_*g*_) are changed between 0.5 and 1.5. (J–N) Mutant conditions: (J) *fra* (*a*_*g*_ = 0), (K) *unc5* (*r*_*g*_ = 0), (L) *slit* (*g*_*r*_ = 0), (M) *Netrin* mutant (*n*_*a*_ = 0) and Netrin expression in glia (*g*_*a*_ = 0.1). See also Figure S1.

We and others have previously reported that Slit-Robo signaling is required for the boundary formation in the fly optic lobe (Tayler et al., 2004, Suzuki et al., 2016). To investigate how Slit/Robo and Netrin, two major axon guidance pathways, regulate the boundary formation, we propose a simple mathematical model that describes their mutual interaction via Slit and Netrin signaling pathways by simply focusing on neuron and glia. In general, Slit always causes repulsion upon binding to Robo receptors, which means glia repel neurons (Figure 4A). In contrast, Netrin signaling regulates either attraction or repulsion. Fra and Unc5 are known as an attractive and repulsive receptor, respectively (Keleman and Dickson, 2001, Timofeev et al., 2012). However, the lamina glial cells express both Fra and Unc5, and therefore it is not clear if Netrin acts as an attractant or a repellent in the developing fly optic lobe. In this situation, Netrin function could be switched depending on its concentration. According to the results of *in vitro* culture experiments, Netrin may act as an attractant when its concentration is low, whereas it may act as a repellent when its concentration is high (Taylor et al., 2015).

Based on this idea, we formulated a mathematical model of boundary formation by Slit and Netrin (Figure 4B). We assume that the interaction between IPC cells and lamina glia is more important compared with that between OPC cells and lamina glia, because IPC cells including IPC NBs always invaded the OPC by penetrating through the lamina glial cells in various mutant backgrounds (Figure 2). In addition, lamina glia-specific knockdown of *unc5* caused the similar boundary defects (Figures 3J–3L). For simplicity, we focused only on the relationship between IPC neuronal cells (including NBs) and lamina glial cells, and ignored OPC- and GPC-derived neurons. We assume that *slit*, *fra*, and *unc5* are expressed in glial cells and that *netrin* and *robo* are expressed in neurons (Figure 4A).

Here, *G*, *N*, *A*, and *R* represent the density of glia, neuron, Netrin, and Slit, respectively (Figure 4B). The changes in the distributions of neuron and glia are calculated with the initial condition in which the two cell types form partially overlapping but separated clusters (Figure 4D). Since *NetB-myc* and *slit-LacZ* signals were undetectable in neurons and glial cells, respectively, in the early third instar larval stage (data not shown), *A* and *R* are set to 0 as an initial condition. Note that our mathematical model is dimensionless. The distance, time, and density do not directly correspond to the actual units.

We initially compared the difference between the following three conditions, Netrin is an attractant, repellent, and both. When we assume that Netrin always acts as an attractant, neuron and glia are mixed with each other due to glia attraction by neuron (Figure S1A). In contrast, if Netrin always acts as a repellent, neuron and glia are separated by a gap between them due to glia repulsion by neuron (Figure S1B). Thus, if Netrin is a simple attractant or repellent, the sharp boundary cannot be established. We next tested the third condition in which the attraction and repulsion of glia by Netrin proportionally change according to Netrin concentration based on the results of *in vitro* culture study (Figure 4C) (Taylor et al., 2015). Intriguingly, neuron and glia show distinct domains with a very small overlap and a short distance forming a sharp boundary (Figure 4E), suggesting that the above-mentioned assumptions are sufficient to explain the boundary formation. The formation of the sharp boundary can be explained by the following dual functions of Netrin. When the peaks of neuron and glia are distant, a low level of Netrin causes the attraction of glia toward neuron (Figure 4F). When they are close to each other with an overlap, a high level of Netrin causes the repulsion of glia from the neuron cluster, whereas a low level of Netrin still causes attraction of the glia cluster (Figure 4G).

We tested the robustness of this result in different parameter sets for migration speed, diffusion speed, and degree of attraction and repulsion by plotting the degree of overlap and the distance between neuron and glia (Figures 4H and 4I). Here, we define that the two clusters of cells form a sharp boundary when their overlap and distance are sufficiently small. The white dotted lines encircle the area in which the degree of overlap is less than 1.2 based on the left panels, whereas the black dotted lines indicate the area in which the distance between two cell clusters is less than 3.5 based on the right panels. These threshold values were chosen according to the control result (Figure 4E). The red lines indicate the intersections between white and black lines showing the range of sharp boundary formation. In the first test, the speed of cell migration and ligand diffusion were changed (0.5 ≤ *a*_*g*_ = *r*_*g*_ = *r*_*n*_ ≤ 1.5 and 5 ≤ *d*_*a*_ = *d*_*r*_ ≤ 20, respectively; Figure 4H). Second, the strength of attraction and repulsion by Netrin were changed (0.5 ≤ *a*_*g*_ ≤ 1.5 and 0.5 ≤ *r*_*g*_ ≤ 1.5, respectively; Figure 4I). These results suggest that cell migration needs to be significantly slower than ligand diffusion and that attraction and repulsion should be balanced to form a sharp boundary. Both these conditions are biologically plausible.

We subsequently asked what happens in conditions that mimic various mutant backgrounds (Figures 4J–4N). In the mutant conditions for *unc5* (*r*_*g*_ = 0) and *slit* (*g*_*r*_ = 0), neuron and glia show significant overlaps (Figures 4K and 4L). These situations may correspond to the invasion of IPC cells into the OPC through the glial cells (Figure 2). At the glia-IPC boundary, there are many IPC NBs, whereas there is no OPC NB at the glia-OPC boundary (Figure 2E). The presence of IPC NBs near the boundary may explain the selective invasion of IPC cells into the OPC, because active proliferation of IPC NBs located within the lamina glia or OPC area would further enhance their invasion. Indeed, the invading IPC cells contain NBs in mutant backgrounds (Figures 2F–2H).

In contrast, large gaps are formed between neuron and glia in the mutant conditions for *fra* (*a*_*g*_ = 0) and *Netrin* (*n*_*a*_ = 0; Figures 4J and 4M). These outcomes can be explained by the lack of glia attraction by Netrin. A similar gap is found when Netrin is ectopically expressed in glia (*g*_*a*_ = 0.1; Figure 4N). An increase in *A* induced by ectopic Netrin production might reduce its attraction (and enhance its repulsion), which eventually causes a large gap between neuron and glia. However, in the real brain tissue, this kind of gap containing no cell does not exist. Surrounding cells would penetrate to fill the gap. We speculate that IPC NBs are somehow forced to fill the gap and eventually invade the OPC through the lamina glia layer *in vivo* (Figure 2). This assumption needs to be validated in the future study. Although our model does not directly demonstrate the boundary defects found in *fra* and *NetAB* mutants, the switch between attraction and repulsion in Netrin signaling clearly explain the mechanism of the boundary formation.

### Netrin Signaling Dysfunction Disrupts the Medulla-Lobula Complex Boundary in the Adult

**Figure 5:**
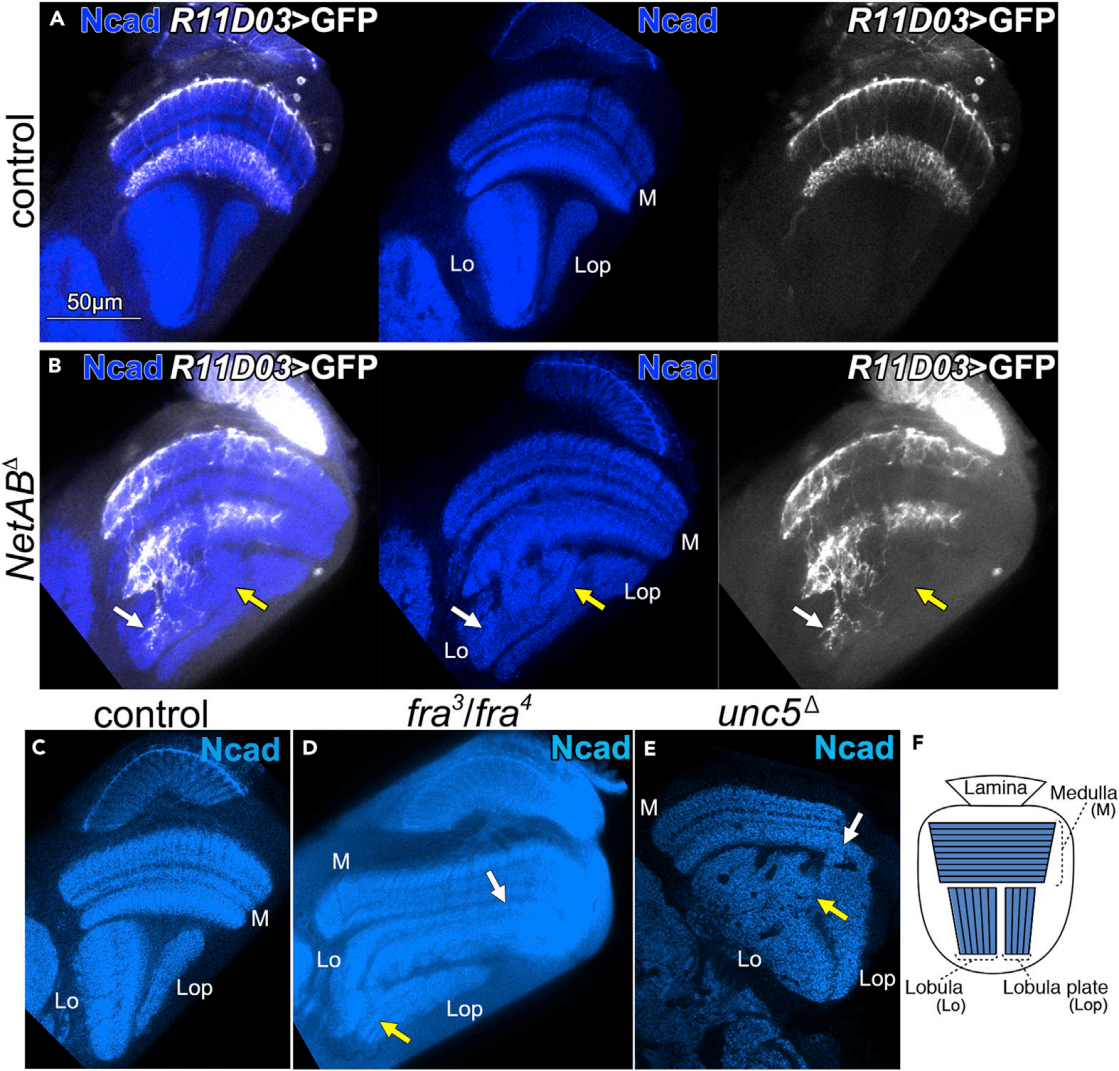
Netrin Signaling Regulates the Boundary Formation between Neuropils in the Adult Optic Lobe (A–E) The adult medulla (M), lobula (Lo), and lobula plate (Lop) are visualized with anti-Ncad antibody (blue). Lateral to the top, anterior to the left. (A and B) Lawf2 neurons are visualized by *R11D03-Gal4 UAS-IVS-GFP* (white). (A) Control. (B) In the *NetAB* mutant, Lawf2 neurons innervate the lobula region (white arrows). The boundaries between the medulla and lobula, and the lobula and lobula plate are disrupted (yellow arrows). (C) Control. In *fra* (D) and *unc5* (E) mutant brains, the medulla-lobula (white arrows) and the lobula-lobula plate boundaries (yellow arrows) are disrupted. (F) Schematic drawing of the adult optic lobe.

Netrin signaling suppression caused disordered arrangement of the medulla neurons and intrusion of IPC cells into the OPC in the larval brain (Figures 2 and 3). We examined the effects of these early defects on the structure of the adult optic lobe. Note that the medulla, lobula, and lobula plate are 90° rotated in a clockwise manner compared with the larval stage (compare Figures 1A3 and 5F). Lamina wide field 2 (Lawf2) neurons project their dendrites throughout the medulla (from layer M1 to layers M9-10 of the medulla) and can be used as a specific marker for the structure of the medulla (Figure 5A) (Hasegawa et al., 2011, Tuthill et al., 2014, Suzuki et al., 2016). Lawf2 neurons visualized by *R11D03-Gal4 UAS-IVS-CD8GFP* do not project to the lobula and lobula plate in control brains (Figure 5A) (Tuthill et al., 2014). In contrast, Lawf2 processes projected to the lobula region (Figure 5B, white arrow; n = 9/14), and the medulla and lobula were obviously intermingled as visualized by Ncad staining in *NetAB*^*Δ*^ brains (Figure 5B; n = 43/67). In addition, the lobula and lobula plate were incompletely separated, and the border between these ganglia was vague in *NetAB*^*Δ*^ brains (Figure 5B, yellow arrow; n = 27/31). Similar disorganization was also observed in *fra*^*3*^/*fra*^*4*^ and *unc5*^*Δ*^ brains (Figures 5C–5E). The medulla and lobula were combined (Figures 5D and 5E; white arrows), and the lobula complex was incompletely separated (Figures 5D and 5E; yellow arrows). Thus, Netrin signaling is required for the compartmentalization between the medulla, lobula, and lobula plate.

## Discussion

The brain is subdivided into multiple distinct regions that consist of many different types of neurons, and each region plays unique roles to carry out complex high-order functions. During development, the brain is subdivided into individual compartments that are defined by sharp borders that inhibit cell migration between different compartments to prohibit the mixing of cells and to contribute to the functional specification of each domain. In the present study, we demonstrate that Netrin signaling is essential for establishing the sharp border between the OPC and IPC in the optic lobe of *Drosophila*. Although we and another group previously reported that Slit-Robo signaling is involved in the regulation of the border formation between the OPC and IPC (Suzuki et al., 2016) and also between the lamina and IPC (Tayler et al., 2004), Netrin signaling is also required for the formation of these borders (Figure 2) and for proper organization of the adult optic lobe (Figure 5). The importance of axon guidance signaling, especially Ephrin-Eph signaling, in border formation has been emphasized in vertebrate brain development (Xu et al., 1995, Xu et al., 1999, Batlle and Wilkinson, 2012, Cayuso et al., 2015). The present study reveals that multiple regulatory mechanisms establish the compartmental subdivision in brains from invertebrates to vertebrates.

### Molecular Mechanisms of Netrin Signaling-Mediated Regulation of Border Formation between OPC and IPC

We found that dysfunction of Netrin signaling caused severe defects in compartmental subdivision of the fly visual center. In *NetAB*, *fra*, and *unc5* mutant brains, IPC cells were strikingly extruded, which results in incomplete separation of the medulla, lobula, and lobula plate in the adult optic lobe (Figure 5). Because the IPC produces lobula and lobula plate neurons, it is plausible that the early defects in the boundary between the OPC and IPC eventually cause the boundary defects between the medulla and the lobula complex.

Our expression analyses indicate that both Fra and Unc5 are localized in the GPC-derived neurons and the lamina glial cells (Figures 1E and 1F), suggesting that Netrin signaling in these neurons and/or lamina glial cells is essential. Although neuron-specific suppression of Netrin signaling also caused IPC extrusion (data not shown), suppression of Netrin signaling in a subset of lamina glial cells was sufficient to disrupt the border (Figures 3J and 3K), suggesting that Netrin signaling in the lamina glial cells needs to be activated to establish the proper arrangement of lamina glial cells and proper compartmental subdivision of the visual center.

Netrin signaling is broadly accepted as a classic guidance signaling pathway (Kennedy et al., 1994, Serafini et al., 1994, Kolodziej et al., 1996, Keleman and Dickson, 2001, Brankatschk and Dickson, 2006, Timofeev et al., 2012), and the arrangement of lamina glial cells can be regulated by its attractive or repulsive activity. It is possible that the disordered distribution of lamina glial cells causes the failure of the compartmental subdivision. Lamina glial cells are neatly arranged within the lamina, and their glial fibers surround the IPC via Netrin-mediated cell attraction or repulsion. This raises a possibility that this glial enclosure contributes to the maintenance of sharp borders around the IPC. This enclosure itself is likely to be formed in the absence of Netrin signaling because the ectopic IPC cells in *NetAB* and *fra* mutants were also surrounded by glial cells (Figures 2J and 3K).

DCC was initially identified as a factor that is deleted in colorectal carcinoma and has been thought to be related to cancer metastasis (Keino-Masu et al., 1996, Rodrigues et al., 2007). Although it has been demonstrated that DCC controls apoptosis induction in *p53*-deficient tumor cells, the mechanism of metastasis caused by the DCC mutant remains unclear (Krimpenfort et al., 2012). Since the mutant of Fra, a fly DCC homologue, causes penetration of IPC cells into neighboring compartments, future studies based on our results may be able to address the mechanism of cancer metastasis found in patients carrying DCC mutations.

### The Switch between Attraction and Repulsion Found in Various Guidance Molecules

According to our mathematical model, the switch between attractant and repellent of Netrin at least partially explains its role in the boundary formation. Although it is technically very difficult to prove if the switch indeed happens *in vivo*, Netrin-1 has been demonstrated to have similar switching functions by *in vitro* culture experiments (Taylor et al., 2015). In addition, the structural analysis of the Netrin1-DCC complex revealed that Netrin binds to two DCC molecules and most likely acts as an attractant when its concentration is low, whereas it binds to one DCC at high concentration (Finci et al., 2014). At higher Netrin concentration, Unc5A may replace DCC to switch from attraction to repulsion. It has been demonstrated that Unc5 is able to regulate repulsion in the absence of Fra in the fly embryonic nervous system (Keleman and Dickson, 2001). Since both Fra and Unc5 are expressed in lamina glial cells in the fly optic lobe, the above-mentioned findings are consistent with the binary function of Netrin assumed in our mathematical model. Since BDNF also shows a similar switching function (Mai et al., 2009), similar strategies may be used in many other biological systems.

Recent findings challenge the classical view of Netrin-dependent long-range attraction in the commissural axon guidance (Dominici et al., 2017, Varadarajan et al., 2017, Yamauchi et al., 2017). In addition, it was shown that Netrin signaling is not required for long-range attraction but promotes adhesion to the target layer (Akin and Zipursky, 2016). However, it is still possible to assume short-range attraction and repulsion by Netrin signaling. Indeed, the phase diagram in Figure 4H shows that small diffusion coefficients of the ligands are compatible with sharp boundary formation when the migration coefficients are small.

### An Interrelationship between Netrin Signaling and Slit-Robo Pathways in Compartmental Subdivision

The present results and a previous report (Tayler et al., 2004) suggest that lamina glial cells regulate the integrated development of each ganglion in the visual center. An important role of lamina glial cells as a source of axon guidance ligands for the formation of the border between the lamina and the IPC has been discussed (Tayler et al., 2004). We conducted glial-cell-specific knockdown of Slit and observed the ectopic appearance of IPC cells in OPC upon induction of *sli RNAi* under the control of *repo-Gal4* or *R25A01-Gal4* (Figure 3D and data not shown). This result suggests that the lamina glial cells are the essential sources of Slit required for the compartmental subdivision. Thus, the lamina glial cells play key roles in controlling the compartmental subdivision by activating Netrin signaling as well as by producing Slit.

In addition to the lamina glial cells, Netrin signaling may also be directly linked with the Slit-Robo pathway in the GPC-derived medulla neurons, given that *fra*, *unc5*, and *sli* are co-expressed in these cells (Figure 1) (Suzuki et al., 2016). Indeed, inactivation of Netrin signaling in neurons also caused the boundary defects (data not shown). A defect in either one of the signaling pathways disrupts the compartmental boundary, implying that both of these signaling systems are indispensable for the compartmental subdivision in the fly optic lobe. The idea that Netrin signaling activates the transcription of *sli* in the lamina glial cells and GPC-derived neurons is attractive because Fra has been shown to act as a transcription factor (Neuhaus-Follini and Bashaw, 2015). However, we have not yet been able to observe such a serial relationship between these two signaling systems.

Nevertheless, Netrin and Slit pathways are broadly conserved from invertebrates to vertebrates. It would be interesting to investigate the details of the molecular mechanisms of the Netrin and Slit-Robo dual regulation system during boundary formation in the brain.

### Limitations of the Study

Although we propose that a simple combination of Slit-dependent repulsion and dual functions of Netrin (an attractant when its concentration is low and a repellent when its concentration is high) is sufficient to explain their roles in boundary formation, our mathematical model is very simplified from the real phenomenon found in the fly brain. Further improvement of the mathematical model and biological experiments will be necessary to address how the mechanism proposed in this study can be applied to developing organisms *in vivo*.

## Methods

All methods can be found in the accompanying Transparent Methods supplemental file.

## Supplemental Information

Document S1. Transparent Methods and Figure S1
