# Supplementary material for: Netrin Signaling Defines the Regional Border in the Drosophila Visual Center
Source: iScience. 2018 Sep 28;8:148–60. doi: 10.1016/j.isci.2018.09.021 (PMC6187055; doi:10.1016/j.isci.2018.09.021)
Supplement: Document S1. Transparent Methods and Figure S1 [file mmc1.pdf]

ISCI, Volume *8*

Supplemental Information

Netrin Signaling Defines the Regional Border in the *Drosophila* Visual Center

Takumi Suzuki, Chuyan Liu, Satoru Kato, Kohei Nishimura, Hiroki Takechi, Tetsuo Yasugi, Rie Takayama, Satoko Hakeda-Suzuki, Takashi Suzuki, and Makoto Sato

SUPPLEMENTAL FIGURES

Figure S1

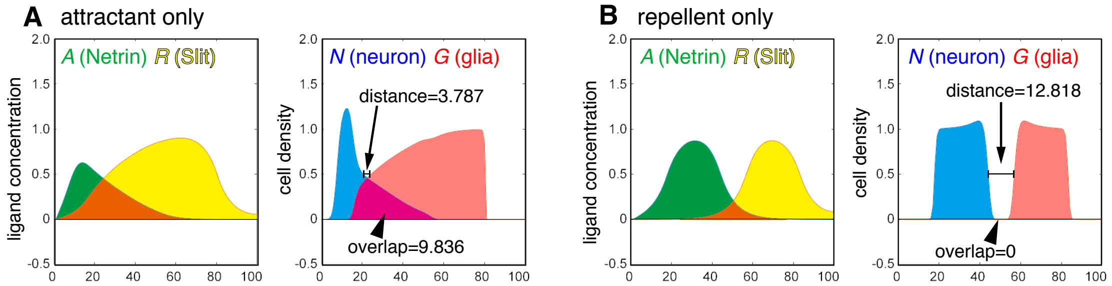

$$\frac{\partial G}{\partial t} = -a_g \nabla \cdot (G \nabla A) + p_g (C_{max} - G - N) G \qquad \frac{\partial G}{\partial t} = r_g \nabla \cdot (G \nabla A) + p_g (C_{max} - G - N) G$$

Figure S1. Numerical results of attractant only and repellent only conditions, Related to Figure 4.

(A) Netrin always acts as an attractant. (B) Netrin always acts as a repellent. Equations of *G* are shown at the bottom.

TRANSPARENT METHODS

Key Resources Table

REAGENT or RESOURCE	SOURCE	IDENTIFIER
Antibodies		
guinea pig anti-Bsh (1:1600)	Hasegawa et al., 2011	N/A
rabbit anti-Fra (1:1000)	Kolodziej et al., 1996	N/A
mouse anti-LacZ (1: 250)	Promega	
chick anti-LacZ (1:1000)	Abcam	
mouse anti-GFP (1:400)	clontech	
rabbit anti-c-Myc (1:100)		
Rabbit anti-NetA (1:200)	University of Mainz	Benjamin Altenhein
rabbit anti-NetB (1:200)	University of Mainz	Benjamin Altenhein
rabbit anti-Unc5 (1:200)	University of Mainz	Benjamin Altenhein
Guinea pig anti-Dpn (1:1000)	Washington University	James Skeath
mouse anti-Eya (1:8)	Developmental Studies Hybridoma Bank (DSHB)	AB_528232
mouse anti-Repo (1:10)	DSHB	AB_528448
rat anti-Ncad (1:20)	DSHB	AB_528121
anti-Fas3 (1:10)	DSHB	AB_528238
anti-guinea pig Cy5 (1:200)	Jackson ImmunoResearch Laboratories	706-175-148
anti-guinea pig Alexa 647 (1:200)	Jackson ImmunoResearch Laboratories	706-605-148
anti-mouse Cy3 (1:100)	Jackson ImmunoResearch Laboratories	715-165-151
anti-mouse Cy5 (1:200)	Jackson ImmunoResearch Laboratories	715-175-151
anti-mouse FITC (1:200)	Jackson ImmunoResearch Laboratories	715-096-151
anti-rat Dylight 649 (1:200)	Jackson ImmunoResearch Laboratories	112-495-175

anti-rat Cy5 (1:200)	Jackson ImmunoResearch Laboratories	712-175-153
anti-rabbit FITC (1:200)	Jackson ImmunoResearch Laboratories	711-095-152
anti-chick Cy3 (1:100)	Jackson ImmunoResearch Laboratories	703-165-155
anti-chick Alexa 647 (1:200)	Jackson ImmunoResearch Laboratories	703-606-155
anti-rabbit Alexa 546 (1:100)	Invitrogen	A-11035
Experimental Models: Organisms/Strains		
UAS-CD8GFP	Bloomington *Drosophila* Stock Center (BDSC)	5130, 5136, 5137
UAS-IVS-CD8GFP	BDSC	32186
UAS-dicer2	BDSC	24650, 36510
R11D03-Gal4	BDSC	48453
R25A01-Gal4	BDSC	49102
dll-Gal4		
omb-Gal4		
repo-Gal4	BDSC	7415
$sli^{JF01228}$	BDSC	31467
$sli^{JF01229}$	BDSC	31468
sli^{GD5822}	Vienna Drosophila Resource Center (VDRC)	v20210
sli^{2}	BDSC	3266
sli^{dui}	Massachusetts Institute of Technology	Paul A. Garrity
$NetAB^{\Delta}$	Janelia Research Campus	Barry J. Dickson
$NetA^{\Delta}$	Janelia Research Campus	Barry J. Dickson
$NetB^{\Delta}$	Janelia Research Campus	Barry J. Dickson
NetB-myc	Janelia Research Campus	Barry J. Dickson
$fra\text{-}lacZ^{SH0030}$	Kyoto Stock Center	122067
fra^{3}	BDSC	8813
fra^{4}	BDSC	8743
$UAS\text{-}fra^{HMS01147}$	BDSC	40826

UAS-unc5^{KK102074}	VDRC	v110155
UAS-unc5^{GD3510}	VDRC	v8138
unc5$^{\Delta}$	this study	N/A
Oligonucleotides		
See method details		
Recombinant DNA		
See method details		
Software and Algorithms		
ZEN image browser	Zeiss	
Photoshop	Adobe	

CRISPR/Cas9-mediated mutagenesis

unc5$^{\Delta}$, a novel *unc5* null allele, was generated by CRISPR/Cas9 technology (Chen et al., 2014; Kondo and Ueda, 2013). Two gRNA vectors (pBFv-U6.2) that recognize the sequences immediately downstream and upstream of the translational start and stop sites, respectively (GCTGAAGCTTAACCAGCAGGAGG and GACATCATAGTTGAAACCATAGG), were injected to eggs carrying *vas-Cas9* (BDSC 55821). A large deletion that removes almost all of the *unc5* ORF was confirmed by sequencing (translational start site-**ATG**GCGGTGATTAATAAAGCCGGAAATGTGATTGCCCTCCT – break point – CATAGGCCCTTTGTGGATT**TAA** -translational stop site).

Immunohistochemistry

Immunohistochemistry was performed as described (Hasegawa et al., 2011). Confocal images were acquired using Zeiss LSM510 or LSM880, and were processed using Zeiss ZEN image browser and Adobe Photoshop. In situ hybridization was performed as described previously [15]. The boundary defects were quantified by comparing the number of brains showing abnormal fusion and/or disruption of the neuropils as visualized by Fas3 and Ncad staining.

Mathematical modeling

The differential equations were calculated using the explicit finite difference method

with the zero-flux boundary condition in one dimension ($1 \leq x \leq 100$). The mesh size and time step size are 1 and 0.01, respectively (dx=1, dt=0.01). The upwind differencing scheme was used to calculate the advection terms. G, N, A and R represent the density of glia, neuron, Netrin and Slit, respectively (Fig. 4B). As an initial condition, the two cell types form partially overlapping but separated clusters (Fig. 4D; A=R=0). The rate of change in A is influenced by its diffusion (d_aA), degradation (k_aA) and production by neuron (n_aN) and glia (g_aG). Similarly, the rate of change in R is influenced by its diffusion (d_rR), degradation (k_rR) and production by glia (g_rG). The attraction and repulsion of neuron (N) and glia (G) are formulated according to the Keller-Segal model of chemotaxis. Since the areas of neuron and glia significantly expand during larval development, logistic growth terms are included so that the maximum cell density becomes C_{max}. Thus, the rate of change in N is influenced by its repulsion by Slit $r_n\nabla\cdot(N\nabla R)$ and by its growth ($p_n(C_{max}$-G-$N)N$). We assume that the coefficient for the attraction and repulsion of glia (G) by Netrin (A) proportionally changes according to Netrin concentration as shown in Fig. 4C. Namely, the attraction coefficient becomes a_g when A=0, while the repulsion coefficient becomes r_g when A_{max}, the upper limit of A. Thus, the rate of change in G is influenced by its attraction and repulsion by Netrin $((r_g+a_g)A/A_{max}-a_g)\nabla\cdot(G\nabla A)$ and by its growth ($p_g(C_{max}$-G-$N)G$). Constant attraction and constant repulsion of glia by Netrin were calculated by $-a_g\nabla\cdot(G\nabla A)$ and $r_g\nabla\cdot(G\nabla A)$, respectively (Fig. S1).

We assume that the corresponding parameters are largely equivalent between Netrin and Slit signalings and between neuron and glia. The diffusion coefficients of the ligands (d_a=d_r=10) are significantly greater than the migration coefficients of the cells (r_n=r_g=a_g=1). The production and degradation rates of ligands are set to modest values to stabilize the ligand distributions (k_a=k_r=n_a=g_r=0.2). Netrin is not produced in the glial cells except for the ectopic Netrin condition (g_a=0). For simplicity, the maximum values for cell density (C_{max}) and ligand concentrations (A_{max} and R_{max}) are set to 1. A and R do not exceed 1.0 in our parameter settings. To focus on the roles of the attraction and repulsion, the effects of cell growth are limited (p_n=p_g=0.01). The initial distributions of N and G are as shown in Fig. 4D (A=R=0). The following results are based on the above settings at t=100,000. The overlap between neuron and glia is the total area in which G>0 and N>0. Since the peak values of G and N tend to be C_{max}, the distance between neuron and glia is the minimal distance between the points of G=N=C_{max}/2=0.5.

SUPPLEMENTAL REFERENCES

Kondo, S., and Ueda, R. (2013). Highly improved gene targeting by germline-specific Cas9 expression in Drosophila. Genetics *195*, 715-721.

Chen, X., Xu, F., Zhu, C., Ji, J., Zhou, X., Feng, X., and Guang, S. (2014). Dual sgRNA-directed gene knockout using CRISPR/Cas9 technology in Caenorhabditis elegans. Sci. Rep. *4*, 7581.

Hasegawa, E., Kitada, Y., Kaido, M., Takayama, R., Awasaki, T., Tabata, T., and Sato, M. (2011). Concentric zones, cell migration and neuronal circuits in the Drosophila visual center. Development *138*, 983-993.
